# Parenting Stress in Households Experiencing Food Insecurity: Mental Health as a Mediator?

**DOI:** 10.1007/s10995-025-04131-5

**Published:** 2025-07-21

**Authors:** Katherine Engel

**Affiliations:** School of Public Affairs, American University, Kerwin Hall, 4400 Massachusetts Ave NW, Washington, DC 20016 USA

**Keywords:** Food insecurity, Mental health, Socioeconomic disparities, Parenting stress

## Abstract

**Objectives:**

To examine associations between food insecurity and parenting stress and assess the extent to which parent and child mental health explain these associations.

**Methods:**

Cross-sectional data from the 2016–2019 National Survey of Children’s Health (*N* = 72,763) were pooled to compare parenting stress between households experiencing different levels of food insecurity. Tests were then performed to determine whether parent and child mental health mediates the association between food insecurity and parenting stress.

**Results:**

Parents in households experiencing mild food insecurity had parenting stress scores that were 0.23 standard deviations higher than parents in food secure households. These parents were also 1.23% points (161.84%) more likely to report handling the demands of parenting poorly compared to parents in food secure households. The association between parenting stress and food insecurity increased in magnitude with more severe household food insecurity; parents in households experiencing moderate-to-severe food insecurity had parenting stress scores that were 0.46 standard deviations higher than parents in food secure households, and these parents were 4.3% points (565.79%) more likely to report handling the demands of parenting poorly compared to parents in food secure households. Differences in child and parent mental health explained only some of the identified disparities in parenting stress.

**Supplementary Information:**

The online version contains supplementary material available at 10.1007/s10995-025-04131-5.

## Significance Statement

What Is Already Known on this Subject

What this Study Adds

Children in food insecure households are at risk for experiencing adverse health and developmental outcomes, even when they do not directly experience low food intake. One potential explanation is that parenting behaviors and stress are harmed by household level food insecurity. Mental health may play a role in the relationship between food insecurity and parenting, but the direction of these relationships is unclear.

This study investigates whether parent and child mental health mediate the relationship between food insecurity and parenting stress. It thus contributes to the literature on how food insecurity affects family functioning, with implications for reducing disparities in child and parent health.

**Supplementary Information:**

The online version contains supplementary material available at 10.1007/s10995-025-04131-5.

## Introduction

Food insecurity, defined as the economic and social condition of limited or uncertain access to adequate food (Definitions of Food Security, 2022), is a persistent problem in the United States. Concerningly, families with children are especially vulnerable to food insecurity. About 18% of households with children experienced food insecurity in 2023 (Rabbitt et al., 2024). The prevalence of food insecurity among households with children is of particular concern given associations between exposure to food insecurity during childhood and child development; children living in food insecure households face an elevated risk of lower levels of well-being, health and developmental issues, and behavioral problems (Frank et al., 2010; Gallegos et al., 2021; Greder et al., 2017; Thomas et al., 2019), which may affect their educational attainment and health as adults (Cohen et al., 2010; McLeod & Kaiser, 2004).

Although children in food insecure households are often shielded by their parents from reductions in food intake, even when children have sufficient nutrition, household food insecurity adversely affects children’s long-term outcomes, potentially by increasing parenting stress and negatively affecting parenting behavior (Bronte-Tinkew et al., 2007; Fiese & Johnson, 2021; Huang et al., 2010; Marçal, 2022). Parents in food insecure households face cognitively and emotionally taxing decisions about meeting material needs, like paying bills and securing housing as well as which family members’ nutritional needs to satisfy (Marçal, 2022; Pignatiello & Hickman, 2019). These parents must not only navigate the inevitable challenges and stress of raising children but also the added burdens imposed by insufficient resources to meet basic needs. These additional demands can increase mental health problems in parents and children and tax parent-child interactions and family functioning (Marçal, 2022; Yoon et al., 2015).

Previous literature indicates that food insecurity may affect child outcomes through the strong link between parental mental health and child development (Greder et al., 2017; Johnson & Markowitz, 2018; Nagata et al., 2019; Saasa et al., 2021; Ward & Lee, 2022), suggesting that the adverse effects of food insecurity on maternal depression and anxiety may harm parenting behaviors and, subsequently, child development. For example, Greder et al. (2017), Nagata et al. (2019), and Saasa et al. (2021) found that maternal depression mediates associations between food insecurity and child behavioral and developmental problems. These findings are consistent with other literature demonstrating the importance of maternal mental health for child development (Tupper et al., 2020).

**Fig. 1:**
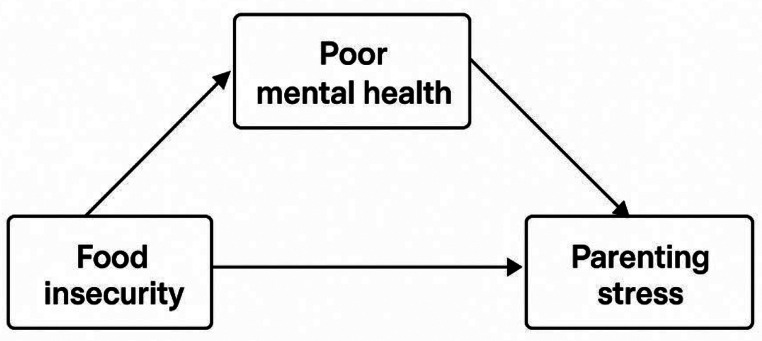
Hypothesized relationship between food insecurity, mental health, and parenting stress

A few studies have directly examined the effect of food insecurity on parenting and provide some additional evidence that mental health may play a role in these associations (Gee & Asim, 2019; Huang et al., 2010; Johnson & Markowitz, 2018). However, the relationships between food insecurity, mental health, and parenting remain unclear, and there is a lack of research identifying the processes through which food insecurity affects parenting. The present study compares parenting stress in households experiencing different levels of food insecurity, examining the extent to which differences in parenting stress between these households can be explained by differences in both parent and child mental health. As shown in Fig. 1, this study tests the hypothesis that food insecurity increases parenting stress, partially through adverse child and parent mental health. Because randomized controlled trials studying food insecurity and parenting are not possible, and opportunities for quasi-experiments are rare, descriptive research examining the role of potential mediators, like mental health, in this relationship is important for understanding pathways through which material hardship and parenting are related, making this study an important contribution to the literature.

## Methods

### Data and Sample

The primary data source for this analysis was the 2016–2019 National Survey of Children’s Health (NCSH) (National Survey of Children’s Health, n.d.). Since 2016, the NCSH has been conducted annually by the US Census Bureau on behalf of the US Department of Health and Human Services Health Resources and Services Administration’s Maternal and Child Health Bureau to generate indicators of the health and well-being of children, families, and communities and of specific healthcare needs. In 2016, 2017, 2018, and 2019, the Census Bureau selected between 170,726 and 364,150 households from their Master Address File across all 50 states and Washington, DC and used a screener to identify households with children aged 0–17 years old. After creating a roster of children within the household, one child per household was randomly selected and an age-specific topical survey was administered to a parent or guardian. Survey weights were included such that estimates are representative of households with children at the state and national level (2016 National Survey of Children’s Health: Methodology Report, 2018). The full sample of respondents for this analysis included those who completed a 2016–2019 NSCH interview about the child randomly selected from their household (*N* = 72,763).^1^

### Dependent Variables

A measure of parenting stress was created using three questions in the NSCH (Shetgiri et al., 2012; Wang et al., 2021). These questions asked how often during the past month the parent felt the child was much harder to care for than most children their age, felt that the child did things that bothered them a lot, and felt angry with the child. The frequency of each of these stresses was measured using a five-point scale, where one indicated that the parent never experienced the feeling, two indicated that they rarely experienced the feeling, three indicated that they sometimes experienced the feeling, four indicated that they usually experienced the feeling, and five indicated that they always experienced the feeling. The internal consistency of these measures of parenting stress was high, with a Cronbach’s alpha score of 0.79, and past work has also used these questions to assess parenting stress (Shetgiri et al., 2012; Wang et al., 2021). The frequency scores were summed across the three questions to create a single measure of parenting stress. This measure was then standardized, such that the mean was zero and standard deviation was one.

As an additional measure, the percent of parents reporting that they were handling the demands of parenting poorly was assessed by constructing a binary variable equal to one if the respondent reported that they thought they were handling the day-to-day demands of raising children not very well or not well at all and zero if they reported that they were handling these demands very well or somewhat well.

### Independent Variables

Following previous work (Baiden et al., 2021), measures of a household’s food security status were constructed.^2^ Respondents who reported that they could always afford to eat good nutritious meals were considered food secure, respondents who indicated they could always afford enough to eat but not always the kinds of food they should eat were considered to be in households experiencing mild food insecurity, and respondents who indicated that they sometimes or often could not afford enough to eat were considered to be in households experiencing moderate-to-severe food insecurity.^3^

To assess the degree to which mental health explains associations between food insecurity and parenting stress, dichotomous measures of both parent and child mental health were used. Parent mental health was measured using a binary variable equal to one if the respondent reported that their mental or emotional health was fair or poor, and zero if they reported their mental or emotional health was excellent, very good, or good, an approach used in previous research (Shetgiri et al., 2012). Two additional binary variables were constructed as measures of child mental health. The first variable equaled one if the parent reported that a doctor or health care provider had told them that the child had anxiety problems and the parent reported the child had anxiety at the time of the interview, and zero if the parent reported that a doctor or other health care provider had not told them that the child had anxiety problems or if a doctor or other health care provider had told them that the child had anxiety problems but the child did not have anxiety at the time of the interview. The second variable equaled one if the parent reported that a doctor or health care provider had told them that the child had depression and the parent reported the child had depression at the time of the interview, and zero if the parent reported that a doctor or other health care provider had not told them that the child had depression or if a doctor or other health care provider had told them that the child had depression but the child did not have depression at the time of the interview.

### Analytic Strategy

Ordinary least squares (OLS) regression was used to estimate the following model to determine whether respondents experiencing food insecurity had higher levels of parenting stress than respondents in food secure households:

1 $$\eqalign{{Y_p} = & \alpha + \beta {\>_1}MildF{I_h} + \beta {\>_2}ModerateSevereF{I_h} \cr& + {\beta _3}{X_h} + \mu {\>_s} + \varphi {\>_y} + {\epsilon _{phsy}} \cr} $$

$\:{Y}_{p}$ was a measure of parenting stress for the parent p. $\:{MildFI}_{h}\:$was a binary variable equal to one if the respondent reported that the household could always afford enough to eat but not always the kinds of nutritious food they should eat and zero otherwise. Similarly, $\:ModerateSevereF{I}_{h}$ was a binary variable equal to one if the respondent reported that the household sometimes or often could not afford enough to eat and zero otherwise. Thus, $\:{\beta\:}_{1}$ and $\:{\beta\:}_{2}$ represented the difference in parenting stress between parents in mildly food insecure and moderately-to-severely food insecure households, respectively, relative to parents in food secure households. $\:{X}_{h}\:$ was a vector of household controls, including Federal Poverty Level (FPL; a common way of assessing household income in relation to needs) percent, the child’s and respondent’s gender (male or female), child race/ethnicity (non-Hispanic White, non-Hispanic Black, Hispanic, Asian, or non-Hispanic other), child and respondent age, the number of children in the household (one, two, three, or four or more), the respondent’s marital status (unmarried or married/living with a partner), the highest level of education among the reported adults in the household (Bachelor’s degree or higher, some college or Associate’s degree, high school, or less than high school), and the respondent’s employment status (whether the respondent was employed at least 50 out of the past 52 weeks). Accordingly, $\:{\beta\:}_{3}$ represented the association between these controls and parenting stress, such that $\:{\beta\:}_{1}$ and $\:{\beta\:}_{2}$ represented the association between food insecurity and parenting stress, net of these demographic factors. State fixed effects, $\:{\mu\:}_{s}$, controlled for time-invariant differences across geographies, and $\:{\varphi\:}_{y}$, year fixed effects controlled for secular time trends.

Due to the cross-sectional nature of the data and lack of exogenous variation, it was not possible to determine whether food insecurity caused parenting stress through poor mental health. However, the following model was estimated to confirm that mental health is significantly and positively associated with food insecurity and thus may plausibly mediate the relationship between food insecurity and parenting stress:

2 $$\eqalign{MentalHealt{h_h} = & \alpha + \beta {\>_1}MildF{I_h} + \beta {\>_2}ModerateSevereF{I_h} \cr& + \beta {\>_3}{X_h} + \mu {\>_s} + \varphi {\>_y} + {\epsilon _{phsy}} \cr} $$

Here,$\:\:MentalHealt{h}_{h}$was a measure of mental health–either a binary variable indicating if the respondent reported that their mental or emotional health was fair or poor, whether their child had anxiety at the time of the interview, and whether their child had depression at the time of the interview–for household h. Aside from this dependent variable, this equation was identical to Eq. (1). Thus, $\:{\beta\:}_{1}\:$and $\:{\beta\:}_{2}$ represented the differences in mental health outcomes between parents in households experiencing mild and moderate-to-severe food insecurity, relative to parents in food secure households. These results are shown in Table A2 and confirm the strong positive association between food insecurity and mental health that is documented in past literature (Afulani et al., 2020; Frongillo et al., 2017; Nagata et al., 2019).

Having established the relationship between food insecurity and mental health, Eq. (3) was used to test whether mental health mediates associations between food insecurity and parenting stress. Specifically, to identify the extent to which mental health explains differences in parenting stress between parents in food secure and food insecure households, controls for mental health were added to Eq. (1):

3 $$\eqalign{{Y_p} = & \alpha \> + \beta {\>_1}MildF{I_h} + \beta {\>_2}ModerateSevereF{I_h} + \beta {\>_3}{X_h} \cr& + \beta {\>_4}MentalHealt{h_h} + \mu {\>_s} + \varphi {\>_y} + {\epsilon _{phsy}} \cr} $$

This model was the same as Eq. (1), except it included a vector of mental health variables, represented by $\:MentalHealt{h}_{h}$, which were outcome variables in Eq. (2). Thus, $\:{\beta\:}_{4}$ represented the association between the mental health measures and parenting stress, net of food security status and other demographic characteristics. The addition of these mental health controls means that $\:{\beta\:}_{1}$ and $\:{\beta\:}_{2}$ represented the difference in parenting stress between parents in mildly food insecure and moderately-to-severely food insecure households, compared to that of parents in food secure households, that is not explained by differences in parent and child mental health. Accordingly, differences between the $\:{\beta\:}_{1}$ and $\:{\beta\:}_{2}$ from Eq. (1) and $\:{\beta\:}_{1}$ and $\:{\beta\:}_{2}$ from Eq. (3) indicated how much of the association between food insecurity and parenting stress was explained by mental health. Seemingly unrelated regression (SUR) was used to test whether the differences between the Eq. (1) and Eq. (3) coefficients were statistically significant (Fiebig, 2003).

## Results

### Descriptive Statistics

About one-third of respondents in the sample reported experiencing some level of food insecurity.^4^ The proportion of the sample experiencing adverse mental health was comparatively low, with about 5% of parents reporting that their mental or emotional health was fair or poor, about 7% reporting that their child had anxiety, and about 3% reporting that their child had depression. Rates of parenting stress were similarly low; the average parenting stress score for the full sample was 0.08, and only 1.3% of parents reported that they were handling the demands of parenting poorly.

However, mental health and parenting stress varied substantially by food security status. As shown in Table 1, without controlling for demographic differences, both children and adults in food insecure households were more likely to have adverse mental health than those in food secure households. Parents in households experiencing mild food insecurity were more than three times and parents in households experiencing moderate-to-severe food insecurity were about nine times more likely to report fair or poor mental health than parents in food secure households. Children in households experiencing mild food insecurity and those in households experience moderate-to-severe food insecurity were also more likely to have anxiety and depression than children in food secure households, with the prevalence of mental health problems increasing in food insecurity severity.

**Table 1:** Descriptive differences in households experiencing food security, mild food insecurity, and moderate-to-severe food insecurity

	Food Secure	Mild Food Insecurity	Moderate-to-Severe Food Insecurity
*Household Characteristics*			
Race/Ethnicity			
Child NH White	65.96%	53.61%***	41.42%***
Child NH Black	10.22%	16.02%***	23.03%***
Child Hispanic	16.39%	22.24%***	25.17%***
Child Asian	1.47%	0.98%*	0.92%
Child NH Other	5.95%	7.15%**	9.46%***
Highest Level of Education in HH			
Less than HS	3.20%	6.01%***	12.58%***
HS or vocational diploma	13.38%	26.89%***	32.15%***
Some college or associate’s degree	18.79%	34.19%***	35.19%***
Bachelor’s degree or higher	64.63%	32.91%***	20.08%***
Respondent was employed 50/past 52 weeks	75.73%	68.03%***	53.47%***
Respondent female	68.83%	79.54%***	87.56%***
Respondent age	40.87	38.97***	39.08
Respondent unmarried	15.00%	28.87%***	45.96%***
Child female	48.87%	48.69%	49.65%
Child age	8.39	8.57	9.15***
Total number of HH children	2.21	2.28***	2.37**
FPL percent	296.98	202.79***	130.83***
*Mental Health*			
Respondent mental health fair or poor	2.38%	8.97%***	22.98%***
	(0.16)	(0.27)	(0.35)
Child currently has anxiety	5.97%	10.31%***	14.01%***
	(0.25)	(0.28)	(0.29)
Child currently has depression	2.18%	4.53%***	9.49%***
	(0.15)	(0.19)	(0.24)
*Parenting Stress*			
Standardized parenting stress score	-0.13	0.01***	0.22***
	(0.98)	(0.95)	(0.99)
Reported handling demands of parenting poorly	0.76%	1.93%***	5.13%***
	(0.09)	(0.13)	(0.18)
Observations	53,691	16,267	2,805

Note: Author’s analysis from the 2016–2019 waves of the NSCH. Estimates are adjusted using NSCH sampling weights and are nationally representative. Table shows means and standard deviations in parentheses. HH is household, NH is non-Hispanic, HS is high school, and FPL is federal poverty level. *p <.05, **p <.01, and ***p <.001 indicates significance of differences between the households indicated and the food secure households based on t-tests

Parents in households experiencing food insecurity also had higher levels of parenting stress than parents in food secure households. Specifically, parents in households experiencing mild food insecurity had parenting stress scores that were 0.14 standard deviations higher than parents in food secure households, and parents in households experiencing moderate-to-severe food insecurity had parenting stress scores that were 0.35 standard deviations higher than parents in food secure households.^5^ Parents in households experiencing mild food insecurity were two and half times more likely to report that they were handling the day-to-day demands of parenting poorly than parents in food secure households. Parents in households experiencing moderate-to-severe food insecurity were more than six times more likely to report that they were handling the day-to-day demands of parenting poorly than parents in food secure households.

## Regression Analysis

Results of the regression analyses are shown in Tables 2 and 3. Table 2 shows the findings for the standardized parenting stress score, and Table 3 shows the findings for the likelihood of reporting handling the demands of parenting poorly. Column 1 of each table shows associations between the outcome variable and food insecurity without any mental health controls (Eq. (1)). Column 2 shows associations between the outcomes and food insecurity with the mental health controls (Eq. (3)).

**Table 2:** Association between food insecurity and standardized parenting stress scores

	(1)	(2)
Mild food insecurity	0.23***	0.16***
	(0.02)	(0.02)
Moderate-to-severe food insecurity	0.46***	0.31***
	(0.05)	(0.04)
Parent mental health fair or poor		0.40***
		(0.04)
Child has anxiety		0.74***
		(0.04)
Child has depression		0.52***
		(0.06)
Year FE	Yes	Yes
State FE	Yes	Yes
Household covariates	Yes	Yes
Constant	-0.43***	-0.42***
	(0.07)	(0.07)
Observations	72,763	72,763

Note: Author’s analysis from the 2016–2019 waves of the NSCH. Models are estimated using ordinary least squares (OLS) regression. Estimates are adjusted using NSCH sampling weights and are nationally representative. *p <.05, **p <.01, and ***p <.001

**Table 3:** Association between food insecurity and the likelihood of reporting handling the demands of parenting poorly

	(1)	(2)
Mild food insecurity	0.012***	0.007**
	(0.00)	(0.00)
Moderate-to-severe food insecurity	0.043***	0.028**
	(0.01)	(0.01)
Parent mental health fair or poor		0.07***
		(0.01)
Child has anxiety		0.01*
		(0.006)
Child has depression		0.03
		(0.015)
Year FE	Yes	Yes
State FE	Yes	Yes
Household covariates	Yes	Yes
Constant	0.00	-0.00
	(0.01)	(0.01)
Observations	72,763	72,763

Note: Author’s analysis from the 2016–2019 waves of the NSCH. Models are estimated using ordinary least squares (OLS) regression. Estimates are adjusted using NSCH sampling weights and are nationally representative. *p <.05, **p <.01, and ***p <.001

As shown in Column 1 of Table 2, these results indicated that parents in households experiencing mild food insecurity had parenting stress scores that were 0.23 standard deviations higher than parents in food secure households. These parents were also 1.2% points (161.84%) more likely to report handling the demands of parenting poorly compared to parents in food secure households, as shown in Table 3 Column 1. The association between parenting stress and food insecurity increased in magnitude with more severe household food insecurity. Specifically, parents in households experiencing moderate-to-severe food insecurity had parenting stress scores that were 0.46 standard deviations higher than parents in food secure households, and these parents were 4.3% points (565.79%) more likely to report handling the demands of parenting poorly compared to parents in food secure households.

As shown in Column 2 of Tables 2 and 3, the coefficients on the food insecurity variables decreased in magnitude but remained statistically significant and substantively meaningful when mental health controls were added. Specifically, parents in households experiencing mild food insecurity had parenting stress scores that were 0.16 standard deviations higher than parents in food secure households. These parents were also 0.7% points (97.37%) more likely to report that they were handling the demands of parenting poorly compared to parents in food secure households. Parents in households experiencing moderate-to-severe food insecurity had parenting stress scores that were 0.31 standard deviations higher than parents in food secure households, and these parents were 2.8% points (361.84%) more likely to be handling the demands of parenting poorly compared to parents in food secure households.

Comparisons across Columns 1 and 2 using SUR indicated that mental health explained some, but not all, of the association between food insecurity and parenting stress. Specifically, mental health explained 0.07 standard deviations of the difference (*p* = .000) in parenting stress scores and 0.50% points (41.67%) of the difference (*p* = .000) in likelihood of reporting handling the demands of parenting poorly between parents in households experiencing mild food insecurity and those in food secure households. Similarly, mental health explained 0.15 standard deviations of the difference (*p* = .000) in parenting stress scores and 1.5% points (34.88%) difference (*p* = .000) in likelihood of reporting handling the demands of parenting poorly between parents in households experiencing moderate-to-severe food insecurity and those in food secure households.^6^

## Discussion

The results of this study are consistent with previous literature indicating that food insecurity is strongly associated with parenting stress (Gee & Asim, 2019; Greder et al., 2017; Huang et al., 2010; Marçal, 2022; Saasa et al., 2021; Tupper et al., 2020). Parents in households experiencing mild food insecurity had parenting stress scores that were 0.23 standard deviations higher than parents in food secure households. These parents were also 161.84% more likely to report they were handling the demands of parenting poorly compared to parents in food secure households. Parents in households experiencing moderate-to-severe food insecurity had parenting stress scores that were 0.46 standard deviations higher than parents in food secure households, and these parents were 565.79% more likely to be handling the demands of parenting poorly compared to parents in food secure households.

This study also expands understanding of the potential pathways through which food insecurity negatively impacts child development; it presents evidence that parent and child mental health explain some of the association between food insecurity and parenting stress. Specifically, the results indicated that mental health explained 0.07–0.15 standard deviations of the difference in parenting stress scores and 34.88-41.67% of the difference in likelihood of reporting handling the demands of parenting poorly between parents in households experiencing food insecurity and those in food secure households. This evidence that mental health mediates the relationship between food insecurity and parenting stress supports the theoretical literature (Conger et al., 1990; Conger & Elder, 1994). It is also consistent with other research suggesting that mental health mediates associations between food insecurity and child behavioral and developmental problems (Greder et al., 2017; Johnson & Markowitz, 2018; Nagata et al., 2019; Saasa et al., 2021) and that indicates that poor mental health, particularly among parents, may be one mechanism through which food insecurity adversely affects child development (Fiese & Johnson, 2021; Huang et al., 2010; Marçal, 2022). However, the findings suggest that mental health is not the sole mechanism through which food insecurity adversely affects parenting stress.

These findings should be interpreted within the context of the limitations of this study. Like other work examining parenting stress (Shetgiri et al., 2012; Wang et al., 2021), this study employed measures of parenting stress and mental health that were reported by parents. The measure of parent mental health may not reflect clinically meaningful differences between these households in terms of mental health diagnoses and healthcare utilization or perceptions of children and other family members. Because the measure of child mental health included medical information on depression and anxiety supplied to parents, it may have more accurately captured meaningful differences. However, because not all children with mental health problems have access to medical services and mental health screening, this measure might have underreported child mental health problems. The measure of food insecurity pertained to household food insecurity and did not identify whether children were food insecure. It is also not comparable to the measure of food insecurity used by USDA, as the NSCH does not include the full food insecurity. However, it is consistent with other measures used in the literature (Baiden et al., 2021). Additionally, the findings are correlational, not causal. Although the theoretical literature provides a foundation for hypothesizing that food insecurity leads to parenting stress, with mental health acting as a mediator, the direction of these associations is not directly tested empirically and is likely bidirectional (Maynard et al., 2018). Mental health controls were added to understand the extent to which mental health explains associations between food insecurity and parenting stress, but these results cannot be interpreted as the causal effect of mental health on parenting stress, because there may be omitted variables that are correlated with mental health. Similarly, the differences in parenting stress that were not explained by mental health cannot be interpreted as the causal effect of food insecurity on parenting stress, because there remain unobserved differences between food secure and food insecure households that may cause differences in parenting stress. Despite these limitations, this study contributes to the literature by investigating a key mechanism through which food insecurity is hypothesized to affect parenting stress (Conger et al., 1990; Conger & Elder, 1994; Greder et al., 2017).

The finding that parent and child mental health explain some of the link between food insecurity and parenting stress has implications for researchers and policymakers interested in reducing disparities in child development and family well-being. Although programs like Supplemental Nutrition Assistance Program (SNAP) and the Special Supplemental Nutrition Program for Women, Infants, and Children (WIC) are explicitly nutrition assistance programs, focused on reducing food insecurity, they may improve family well-being and child development through their indirect effects on mental health and parenting stress. Additionally, the findings highlight that mental health support services, particularly for parents, may benefit families experiencing material hardship like food insecure, potentially reducing inequalities in child development. However, mental health support alone is likely insufficient for eliminating disparities in parenting stress between food secure and food insecure families. Future research and policy should explore the coupling of mental health services and food assistance programs to reduce parenting stress.

## Electronic Supplementary Material

Below is the link to the electronic supplementary material.

Supplementary Material 1

## Data Availability

The National Survey of Children’s Health (NSCH) data are made publicly available by the Maternal and Child Health Bureau at the Health Resources and Services Administration (HRSA). See: https://www.childhealthdata.org/learn-about-the-nsch/NSCH.

## Abbreviations

USDA: United States Department of Agriculature
NSCH: National Survey of Children’s Health

## Footnotes

1 All respondents were, by definition, caregivers, and for the purposes of this analysis, they were all considered parents, although a small number (4.60%) were non-parent guardians or caregivers.

2 The NSCH only collects data on food insecurity at the household level and does not identify whether children are food insecure. As household food insecurity is strongly associated with child development even when children themselves have sufficient food intake (An et al., 2023; Bronte-Tinkew et al., 2007; Eagleton et al., 2022; Gee & Asim, 2019; Greder et al., 2017; Huang et al., 2010; Johnson & Markowitz, 2018; Marçal, 2022; Nagata et al., 2019; Saasa et al., 2021; Ward & Lee, 2022), measuring food security at the household level is appropriate for this analysis.

3 This is not the same measure of food insecurity used by United States Department of Agriculture (USDA), as the NSCH does not include the full food insecurity scales.

4 This rate is higher than estimates published by USDA, likely because the NCSH does not use the full food insecurity scales.

5 Unstandardized means and standard deviations of the parenting stress score and the components of the score are included in Table A1.

6 Predicted values for parenting stress scores and the likelihood of reporting handling the demands of parenting poorly among parents in food secure, mildly food insecure, and moderately-to-severely food insecure households who reported that they and/or the child had mental health concerns and who reported no mental health concerns were also calculated to demonstrate the differences between respondents by food security status and mental health status. These analyses are shown in Figure A1. The appendix also includes a Gelbach Decomposition analysis to try to identify whether child or parent mental health explains more of the association (Gelbach, 2016; Shin, 2023).

## Code Availability

The author will make Stata code available by request.
