# Supplementary material for: Parenting Stress in Households Experiencing Food Insecurity: Mental Health as a Mediator?
Source: Matern Child Health J. 2025 Jul 21;29(9):1244–52. doi: 10.1007/s10995-025-04131-5 (PMC12460468; doi:10.1007/s10995-025-04131-5)
Supplement: Supplementary file 1 — Supplementary Material 1 [file 10995_2025_4131_MOESM1_ESM.docx]

**Appendix**

*Supplemental Tables and Figures*

**Table A1**. Descriptive differences in unstandardized parenting stress in households experiencing food security, mild food insecurity, and moderate-to-severe food insecurity.

|  | Food Secure | Mild Food Insecurity | Moderate-to-Severe Food Insecurity |
| --- | --- | --- | --- |
| Unstandardized parenting stress score | 4.91 | 5.20*** | 5.64*** |
|  | (2.03) | (1.96) | (2.04) |
| Child much harder to care for than most children their age | 1.46 (0.83) | 1.58*** (0.85) | 1.78*** (0.92) |
| Child does things that bother them a lot | 1.69 (0.82) | 1.80*** (0.81) | 2.00*** (0.83) |
| Felt angry with the child | 1.77 | 1.81*** | 1.87*** |
|  | (0.75) | (0.70) | (0.69) |
| Observations | 53,691 | 16,267 | 2,805 |

Note: Author’s analysis from the 2016-2019 waves of the NSCH. Estimates are adjusted using NSCH sampling weights and are nationally representative. Table shows means and standard deviations in parentheses. *p<.05, **p<.01, and ***p<.001 indicates significance of differences between the households indicated and the food secure households based on t-tests.

**Table A2.** Association between food insecurity and mental health.

|  | Parent mental health fair or poor | Child has anxiety | Child has depression |
| --- | --- | --- | --- |
| Mild food insecurity | 0.05*** | 0.05*** | 0.02*** |
|  | (0.006) | (0.005) | (0.003) |
| Moderate-to-severe food insecurity | 0.17*** | 0.08*** | 0.061*** |
|  | (0.015) | (0.011) | (0.011) |
| Year FE | Yes | Yes | Yes |
| State FE | Yes | Yes | Yes |
| Household covariates | Yes | Yes | Yes |
| Constant | 0.085***  (0.019) | -0.042* (0.016) | -0.040***  (0.011) |
|  |  |  |  |
| Observations | 72,763 | 72,763 | 72,763 |

Note: Author’s analysis from the 2016-2019 waves of the NSCH. Models are estimated using ordinary least squares (OLS) regression. Estimates are adjusted using NSCH sampling weights and are nationally representative. *p<.05, **p<.01, and ***p<.001

**Figure A1**. Predicted values of parenting stress by food security status, mental health problems v. no mental health problems.

Note: Author’s analysis from the 2016-2019 waves of the NSCH. Models are estimated using ordinary least squares (OLS) regression. Estimates are adjusted using NSCH sampling weights and are nationally representative. Regressions control for child race/ethnicity, highest level of education attained in the household, federal poverty level percent, parent employment, child and parent age, child and parent gender, total number of household children, parent marital status, and year and state fixed effects. Predicted values are calculated holding all the covariates at their means. No MH Problems indicates that parents reported that their mental health was excellent, very good, or good and the child did not have depression or anxiety at the time of the survey. MH Problems indicates that parents reported that their mental health was fair or poor or the child had depression or anxiety at the time of the survey. *p<.05, **p<.01, and ***p<.001 based on t-tests comparing No MH Problems v. MH Problems. N=72,763.

**Table A3.** Association between food insecurity and the likelihood of reporting handling demands of parenting poorly, alternate model estimation.

|  | (1) | (2) |
| --- | --- | --- |
|  |  |  |
|  |  |  |
| Mild food insecurity | 1.99*** | 1.73*** |
|  | (0.16) | (0.16) |
| Moderate-to-severe food insecurity | 1.92*** | 1.29*** |
|  | (0.23) | (0.26) |
| Parent mental health fair or poor |  | 1.94*** |
|  |  | (0.18) |
| Child has anxiety |  | 1.62** |
|  |  | (0.30) |
| Child has depression |  | 1.64*** |
|  |  | (0.22) |
| Year FE | Yes | Yes |
| State FE | Yes | Yes |
| Household covariates | Yes | Yes |
| Constant | -5.29*** | -5.74*** |
|  | (0.57) | (0.61) |
| Observations | 72,763 | 72,763 |

Note: Author’s analysis from the 2016-2019 waves of the NSCH. Models are estimated using logistic regression. Estimates are adjusted using NSCH sampling weights and are nationally representative. *p<.05, **p<.01, and ***p<.001

*Decomposing Parent and Child Mental Health*

To separately estimate the contribution of parent mental health and child mental health to differences in parenting stress between food secure and food insecure households, this study used the Gelbach Decomposition. This decomposition approach is based on the omitted variables bias formula and addresses the sequencing sensitivity of covariates (Clark & Shi, 2020; Gelbach, 2016; Shin, 2023). In this case, because parent and child mental health are correlated, the order in which the corresponding covariates are added to equation (1) has implications for interpreting the degree to which parent and child mental health separately account for the difference in parenting stress between food secure and food insecure households estimated using equation (3). Results of the Gelbach Decomposition analysis investigating this are shown in Table A4 and Figure A2. Because the strict assumptions of this decomposition only hold for binary “treatment,” this analysis compares households experiencing any food insecurity (mild or moderate-to-severe) to food secure households, so these results are not directly comparable to the main findings.

For standardized parenting stress scores, although the addition of parent and child mental health controls, household characteristics, and year and state fixed effects changed the magnitude of the coefficient on the food insecurity indicator, this aggregate change was not statistically significant. However, the decomposition revealed that, as expected, worse mental health in children and parents was associated with higher parenting stress in food insecure households, relative to food secure households. Although the coefficient on child mental health was larger than that on parent mental health, they were not statistically different, so it is unclear whether child or parent mental health explains more of the association between food security status and parenting stress. For the likelihood of reporting handling the demands of parenting poorly, the addition of parent and child mental health controls, household characteristics, and year and state fixed effects resulted in a statistically significant decrease in the magnitude of the coefficient on the food insecurity variable. This decrease was about one percentage point, suggesting that once these other factors were accounted for in the model, the gap in likelihood of handling the demands of parenting poorly between parents in food secure and food insecure households was more than 40% lower than it appeared in the naïve model. As hypothesized, the addition of the parent and child mental health controls specifically explained much of this decrease, with parent mental health explaining about 2.5 times more of the reduction than child mental health.

**Table A4.** Decomposition of differences in parenting stress between parents in food secure and food insecure households

|  | (1) |
| --- | --- |
| *Standardized parenting stress score* |  |
| Base specification | 0.178*** |
|  | (0.02) |
| Full specification | 0.184*** |
|  | (0.02) |
| Total explained difference (Base-Full) | -0.007 |
|  | (0.01) |
| Contributions to explained difference |  |
| Parent mental health | 0.038*** |
|  | (0.00) |
| Child mental health | 0.054*** |
|  | (0.00) |
| Household characteristics | -0.095*** |
|  | (0.01) |
| Year and State FEs | -0.003** |
|  | (0.00) |
| *Likelihood of handling demands of parenting poorly* |  |
| Base specification | 0.017*** |
|  | (0.00) |
| Full specification | 0.010*** |
|  | (0.00) |
| Total explained difference (Base-Full) | 0.007*** |
|  | (0.00) |
| Contributions to explained difference |  |
| Parent mental health | 0.007*** |
|  | (0.00) |
| Child mental health | 0.002*** |
|  | (0.00) |
| Household characteristics | -0.001* |
|  | (0.00) |
| Year and State FEs | 0.000 |
|  | (0.00) |

Notes: Author’s analysis from the 2016-2019 waves of the NSCH. Estimates are adjusted using NSCH sampling weights and are nationally representative. Base specification is the coefficient on the food insecurity binary variable from regressing the parenting stress outcome variable on just this indicator of food insecurity. Full specification is the coefficient on the food insecurity binary variable from regressing the parenting stress outcome on this indicator of food insecurity, the child and parent mental health variables, the full set of household covariates, and year and state fixed effects. Percent of the explained difference is calculated by dividing the decomposition coefficient on each variable/group of variables by the total explained difference. N=72,763. *p<.05, **p<.01, and ***p<.001

**Figure A2.** Decomposition of differences in parenting stress between parents in food secure and food insecure households

Notes: Author’s analysis from the 2016-2019 waves of the NSCH. Estimates are adjusted using NSCH sampling weights and are nationally representative. Figure shows coefficients and 95% confidence intervals from the Gelbach Decomposition of the gap in parenting stress outcomes between food secure and food insecure households. Total explained difference is the difference in the food insecurity coefficient in a regression model in which the parenting stress variable is regressed on an indicator of food insecurity only (the base specification) and the food insecurity coefficient in a regression model in which the parenting stress variable is regressed on an indicator of food insecurity, the parent and child mental health controls, the full set of household covariates, and year and state fixed effects (the full specification). N=72,763.
